# Effect of a fast-track nursing pathway for emergency percutaneous coronary intervention on clinical outcomes in patients with acute myocardial infarction

**DOI:** 10.3389/fcvm.2025.1679887

**Published:** 2025-11-18

**Authors:** Cai-Yun Bai, Ying Yang, Hui-Yan Xiao, Jin Wang, Kai-Yun Xu

**Affiliations:** 1 Department of Emergency Medicine, Third Affiliated Hospital of Naval Medical University, Shanghai, China; 2 Department of Emergency Medicine and Critical Care Medicine, Marine Corps Hospital, Chaozhou, Guangdong, China

**Keywords:** acute myocardial infarction, percutaneous coronary intervention, fast-track nursing, emergency care, patient satisfaction

## Abstract

**Background:**

Acute myocardial infarction (AMI) requires prompt revascularization to optimize outcomes. This study aimed to evaluate the effect of a structured fast-track nursing pathway on clinical outcomes in patients undergoing emergency percutaneous coronary intervention (PCI).

**Methods:**

This retrospective cohort study included 324 AMI patients admitted through the emergency department between May 2023 and May 2025. Patients treated prior to the implementation of a fast-track nursing pathway (*n* = 156) formed the control group, while those treated after pathway implementation (*n* = 168) formed the observation group. The fast-track pathway included dedicated nursing teams, standardized triage-to-catheterization workflows, expedited monitoring and preparation procedures, and structured patient-family education. Clinical outcomes assessed included time to reperfusion, rescue success rate, left ventricular function [left ventricular ejection fraction (LVEF), left ventricular end-systolic volume (LVESV), and left ventricular end-diastolic volume (LVEDV)], incidence of adverse events, and patient satisfaction.

**Results:**

Compared with the control group, the observation group had significantly shorter resuscitation room time and reperfusion time (*p* < 0.001), and a higher rescue success rate (94% vs. 79%). By day 3, LVEF improvement was significantly greater in the observation group (*p* < 0.001). There were no significant differences in LVESV, LVEDV, or adverse event incidence between groups. The observation group reported higher total satisfaction (96.4% vs. 80.1%, *p* < 0.001).

**Conclusions:**

The fast-track nursing pathway significantly enhanced emergency care efficiency, improved early cardiac recovery, and increased satisfaction without compromising patient safety. These findings support broader adoption of nurse-led process innovations in AMI care.

## 1 Introduction

Acute myocardial infarction (AMI) remains a leading cause of morbidity and mortality worldwide despite significant advances in reperfusion therapy and secondary prevention strategies. Rapid restoration of coronary blood flow through percutaneous coronary intervention (PCI) is the cornerstone of modern AMI management, with a well-documented inverse relationship between door-to-balloon time and infarct size, ventricular remodeling, and long-term survival (1, 2). However, delays in patient transfer, in-hospital processing, and interdisciplinary communication often prolong total ischemic time, thereby diminishing the benefits of timely revascularization. Consequently, healthcare systems have increasingly adopted streamlined protocols, commonly referred to as fast-track pathways, to expedite PCI and improve clinical outcomes (3).

Nursing care plays a pivotal role in the management of emergency PCI for patients with AMI, encompassing early recognition, hemodynamic monitoring, procedural preparation, and post-procedural support. Traditional nursing workflows, often characterized by sequential assessments, redundant documentation, and non-standardized communication channels, may prolong critical intervals between patient arrival and arterial reperfusion (4, 5). A fast-track nursing pathway reorganizes these processes by defining roles in advance, streamlining preparatory steps, and ensuring real-time coordination with catheterization laboratory personnel. Such pathways typically involve early activation of the catheterization team following electrocardiographic confirmation of ST-segment elevation, concurrent completion of consent and pre-procedure checklists, and immediate patient transfer to the catheterization suite under continuous nursing supervision. Emerging evidence indicates that implementing a fast-track nursing pathway can substantially reduce door-to-balloon times, improve adherence to guideline-recommended treatment targets, and enhance surrogate indicators of myocardial salvage (6, 7). Studies conducted in high-volume tertiary centers have demonstrated average reductions of 15–30 min in door-to-balloon intervals after pathway implementation, along with lower peak cardiac biomarker levels and improved left ventricular ejection fraction (LVEF) at discharge. Furthermore, expedited nursing processes may contribute to shorter intensive care unit stays, reduced incidence of heart failure, and lower in-hospital mortality. However, variability in pathway design, differences in institutional resources, and inconsistent staff training remain barriers to widespread implementation, and evidence regarding long-term outcomes is still limited (8, 9).

By delineating the impact of a fast-track nursing pathway on time-sensitive care processes and clinically meaningful endpoints, this study aims to inform best practices in AMI management and promote wider adoption of nursing-led process innovations in acute cardiovascular care. The findings may support future guideline updates and the development of standardized nursing protocols for AMI.

## 2 Methods

### 2.1 Study design

This retrospective study was conducted at our institution between May 2023 and May 2025. Beginning in May 2024, the department formally implemented an emergency PCI fast-track care pathway. A total of 156 patients with AMI who were admitted through the emergency department and underwent emergency PCI from May 2023 to April 2024 comprised the control group (before pathway implementation). The observation group included 168 patients who were admitted and treated under the fast-track nursing care pathway from May 2024 to May 2025. Eligible patients were aged 18 years or older, diagnosed with AMI based on clinical presentation, electrocardiographic changes, and elevated cardiac biomarkers, and had complete clinical and follow-up data. All patients were admitted through the emergency department and underwent emergency PCI. Exclusion criteria included: symptom onset exceeding 12 h without evidence of ongoing ischemia; prior coronary artery bypass grafting (CABG); cardiogenic shock requiring mechanical circulatory support (such as intra-aortic balloon pump or extracorporeal membrane oxygenation) at admission; severe comorbidities with limited life expectancy; contraindications to dual antiplatelet or anticoagulant therapy; pregnancy or lactation; and incomplete medical records. Informed consent was obtained from all subjects and/or their legal guardian(s). The study was reviewed and approved by our hospital's ethics committee and conducted in accordance with relevant guidelines and the Declaration of Helsinki. All data were anonymized before analysis to ensure confidentiality and protect participant privacy.

### 2.2 Nursing interventions

Patients in the control group received conventional emergency nursing care based on standard institutional protocols. In contrast, patients in the observation group were managed using a structured fast-track nursing pathway specifically designed for emergency PCI. This pathway aimed to optimize coordination, minimize time delays, and enhance perioperative safety through standardized procedures. The implementation involved the following components:

1. Establishment of a Dedicated Fast-Track Nursing Team: A specialized emergency PCI nursing team was established, consisting primarily of senior registered nurses with advanced cardiovascular training, along with designated interventional cardiologists. Team responsibilities were clearly delineated following interdisciplinary discussion. All team members received targeted training to ensure familiarity with the emergency PCI fast-track protocol, competency in time-sensitive interventions, and readiness for coordinated response upon patient arrival.
2. Development of a Standardized Emergency PCI Nursing Pathway: Recognizing that nearly 50% of AMI-related deaths occur within the first hour of symptom onset, the nursing pathway was developed based on clinical pathway principles combined with green channel triage strategies. The pathway was designed to streamline processes from emergency room admission to catheterization lab entry, reduce unnecessary procedural steps, shorten reperfusion time, and facilitate rapid decision-making.
3. Reception and Initial Assessment: Upon ED arrival, patients were immediately evaluated by the fast-track nursing team. Reception nurses promptly alerted the interventional team and initiated continuous monitoring of vital signs within 5 min. Electrocardiography (ECG) was completed without delay, and any ST-segment changes or arrhythmias triggered emergency preparation, including defibrillator setup and medication readiness. Intravenous access was established via the left upper extremity to preserve the right radial artery for potential catheterization. Oxygen was administered via mask or nasal cannula at 4–6 L/min. A contrast agent allergy test was conducted within 20 min as per physician orders. Psychological support was provided to reduce anxiety and prevent arrhythmia caused by agitation. Coronary vasodilators and analgesics were administered as prescribed. In parallel, patients and families were educated about PCI using visual aids to enhance understanding and compliance. A designated liaison nurse communicated patient status to the catheterization lab, guided family members through administrative procedures, and explained the surgical procedure to reduce delays caused by hesitancy in signing consent.
4. Intra-Hospital Transport: Within 30 min of arrival, a designated transport nurse from the team escorted the patient to the catheterization laboratory, ensuring continuous ECG monitoring and secure fixation of IV lines and oxygen tubing. A defibrillator was carried as a precaution during transit.
5. Preoperative Preparation in the Catheterization Laboratory: Upon notification from the ED, catheterization lab staff initiated immediate surgical preparation. Required surgical instruments, emergency medications (e.g., lidocaine, epinephrine, atropine), and saline-soaked gauze were prepared and placed in accessible positions. The defibrillator and temporary pacemaker were checked and kept on standby. A designated nurse managed patient handoff and communication with the interventional physician.
6. Intraoperative Nursing Management: PCI was initiated within 35 min of hospital admission. The interventional nurse continuously monitored ECG, invasive blood pressure, and oxygen saturation. Any abnormalities were promptly reported and addressed. Contrast agents were replaced as needed, and resuscitation equipment was prepared in anticipation of potential complications. After sheath removal, radial artery compression was applied using a balloon compression device, maintaining distal pulse and pressure for 6–8 h with gradual deflation every 2 h. Limb immobilization and close monitoring of puncture site and distal circulation were ensured throughout the postoperative period.
7. Discharge and Health Education: Before discharge, patients received individualized education from designated nursing staff, focusing on lifestyle modifications such as smoking cessation, dietary adjustment, regular bowel habits, and physical activity tailored to heart rate and symptom tolerance. Instructions included medication adherence, early recognition of adverse effects, and the importance of timely follow-up. Patients were encouraged to carry emergency medication and seek medical attention if necessary.

### 2.3 Data collection and outcome measures

Clinical data were collected from both the control and observation groups during the acute management and hospitalization period. The following parameters were documented:

1. Emergency Treatment Metrics: Key time intervals and clinical rescue outcomes were recorded, including the length of stay in the resuscitation room, time to myocardial reperfusion (defined as the interval from emergency department arrival to successful coronary revascularization), and overall resuscitation success rate.
2. Cardiac Function Assessment: Cardiac function was evaluated on the day of admission and again on the third day of hospitalization. A Philips EPIQ 7C color Doppler ultrasound system was used for transthoracic echocardiography. Measurements included left ventricular end-diastolic volume (LVEDV), left ventricular end-systolic volume (LVESV), and LVEF, calculated using the biplane Simpson's method in accordance with the American Society of Echocardiography (ASE) guidelines.
3. Adverse Events: All major adverse clinical events occurring during the intervention period were recorded, including infection, cardiac rupture, heart failure, cardiogenic shock, arrhythmias, and all-cause mortality.
4. Patient Satisfaction: Before transfer from the emergency department, family members of the patients were asked to complete a satisfaction questionnaire developed by the department. Responses were categorized into “very satisfied,” “generally satisfied,” and “dissatisfied.” The overall satisfaction rate was calculated as the proportion of “very satisfied” plus “generally satisfied” responses relative to the total number of valid responses.

### 2.4 Statistical analysis

All statistical analyses were performed using SPSS software (version 26.0; IBM Corp., Armonk, NY, USA). Continuous variables were tested for normality using the Kolmogorov–Smirnov test. Variables conforming to a normal distribution were expressed as mean ± standard deviation (SD), and intergroup comparisons were conducted using the independent samples *t*-test. Non-normally distributed data were presented as median (interquartile range) and compared using the Mann–Whitney *U* test. Categorical variables were expressed as frequencies and percentages, and differences between groups were assessed using the chi-square test or Fisher's exact test. These variables included the resuscitation success rate, incidence of adverse events, and overall patient satisfaction rate. Repeated measurements of cardiac function parameters (LVEDV, LVESV, LVEF) at two time points (on admission and at day 3 of hospitalization) were analyzed using repeated measures analysis of variance (ANOVA) to assess both within-group and between-group effects over time. A *p*-value <0.05 was considered statistically significant for all analyses.

## 3 Results

### 3.1 Baseline clinical characteristics

As shown in Table 1, there were no statistically significant differences in baseline demographic or clinical characteristics between the observation group (*n* = 168) and the control group (*n* = 156) (*p* > 0.05 for all). The two groups were comparable in gender distribution, age, onset-to-visit time, and the prevalence of comorbidities such as diabetes, hypertension, and hyperlipidemia. Furthermore, APACHE II scores did not differ significantly between groups, indicating a comparable baseline severity of illness.

**Table 1:** Baseline clinical characteristics of patients with acute myocardial infarction.

Variable	Observation group (*n* = 168)	Control group (*n* = 156)	Statistics (*t*/*χ*^2^)	*p*-Value
Male (%)	64% (108/168)	57% (90/156)	0.480	0.224
Age (years)	57.30 ± 5.29	57.23 ± 4.78	0.125	0.901
Onset-to-visit time (h)	1.04 ± 0.49	1.07 ± 0.52	0.535	0.593
Diabetes (%)	40% (67/168)	39% (61/156)	0.021	0.886
Hypertension (%)	57% (96/168)	52% (81/156)	0.889	0.346
Hyperlipidemia (%)	51% (85/168)	55% (86/156)	0.667	0.414
APACHE II score	19.97 ± 2.08	20.32 ± 2.21	1.469	0.143

CHD, Coronary Heart Disease; APACHE II, Acute Physiology and Chronic Health Evaluation II.

### 3.2 Emergency treatment outcomes

The emergency treatment outcomes for both groups are summarized in Table 2. The observation group demonstrated significantly better performance across all key emergency care indicators compared with the control group (*p* < 0.001 for all). Specifically, the resuscitation room time was significantly shorter in the observation group (47.53 ± 14.58 min) than in the control group (56.44 ± 13.58 min). Likewise, the time to myocardial reperfusion was markedly reduced in the observation group (92.89 ± 17.12 min) compared with the control group (104.39 ± 19.43 min). These findings indicate improved efficiency in emergency care following implementation of the fast-track nursing pathway. Moreover, the rescue success rate was significantly higher in the observation group (94%) than in the control group (79%), reflecting enhanced early clinical stabilization among patients who received fast-track nursing care.

**Table 2:** Comparison of emergency treatment outcomes between the observation and control groups.

Variable	Observation group (*n* = 168)	Control group (*n* = 156)	*t*/*χ*^2^-value	*p*-Value
Resuscitation room time (min)	47.53 ± 14.58	56.44 ± 13.58	5.680	<0.001
Reperfusion time (min)	92.89 ± 17.12	104.39 ± 19.43	5.662	<0.001
Rescue success rate (%)	94% (158/168)	79% (124/156)	15.201	<0.001

Reperfusion Time, time from admission to successful myocardial reperfusion;.

Rescue Success Rate, percentage of patients who achieved clinical stabilization following emergency intervention.

### 3.3 Comparison of left ventricular function

As shown in Table 3, both groups exhibited improvements in cardiac function by day 3 after admission; however, the observation group demonstrated a significantly greater increase in LVEF. On day 1, LVEF values were comparable between groups (50.55 ± 3.20% vs. 49.98 ± 2.80%, *p* = 0.090), whereas by day 3, LVEF was significantly higher in the observation group (54.43 ± 3.32%) compared with the control group (51.36 ± 3.12%) (*p* < 0.001), indicating better recovery of systolic function. No significant differences were found in LVESV or LVEDV at either time point (*p* > 0.05 for all comparisons), suggesting that although both groups showed partial structural recovery, the fast-track nursing pathway was particularly effective in promoting early functional improvement, as evidenced by the greater increase in LVEF.

**Table 3:** Comparison of left ventricular function parameters in AMI patients on Day 1 and Day 3 after admission.

Variable	Observation group (*n* = 168)	Control group (*n* = 156)	*F*-value	*p*-Value
LVEF Day 1 (%)	50.55 ± 3.20	49.98 ± 2.80	1.701	0.090
LVEF Day 3 (%)	54.43 ± 3.32	51.36 ± 3.12	8.561	<0.001
LVESV Day 1 (mL)	68.89 ± 2.26	69.25 ± 2.42	1.385	0.167
LVESV Day 3 (mL)	67.62 ± 3.12	67.33 ± 2.92	0.862	0.389
LVEDV Day 1 (mL)	138.97 ± 26.37	133.92 ± 25.48	1.751	0.081
LVEDV Day 3 (mL)	130.96 ± 22.92	132.54 ± 23.28	0.615	0.539

LVEF, Left Ventricular Ejection Fraction; LVESV, Left Ventricular End-Systolic Volume; LVEDV, Left Ventricular End-Diastolic Volume; AMI, Acute Myocardial Infarction.

### 3.4 Incidence of adverse events

The incidence of adverse events during hospitalization is summarized in Table 4. Overall, the total complication rates were low and comparable between the two groups. The observation group had an adverse event rate of 7.7% (13/168), identical to that of the control group (12/156, 7.7%). Statistical analysis revealed no significant difference between groups (*χ*^2^ = 0.615, *p* = 0.539). Individual complications, including heart failure, arrhythmia, infection, and cardiac rupture, were infrequent in both groups. The most common adverse event was arrhythmia, occurring in 4.2% of patients in the observation group and 1.9% in the control group. Heart failure and infection occurred at similarly low rates, and no cases of cardiogenic shock or cardiac rupture were reported in either group.

**Table 4:** Incidence of adverse events in patients with acute myocardial infarction.

Adverse event	Observation group (*n* = 168)	Control group (*n* = 156)	*χ*^2^-value	*p*-Value
Heart failure	3 (1.8%)	3 (1.9%)	—	—
Arrhythmia	7 (4.2%)	3 (1.9%)	—	—
Cardiogenic shock	0 (0.0%)	0 (0.0%)	—	—
Infection	3 (1.8%)	6 (3.8%)	—	—
Cardiac rupture	0 (0.0%)	0 (0.0%)	—	—
Total incidence	13 (7.7%)	12 (7.7%)	0.615	0.539

LVEF, Left Ventricular Ejection Fraction; LVESV, Left Ventricular End-Systolic Volume; LVEDV, Left Ventricular End-Diastolic Volume; AMI, Acute Myocardial Infarction.

### 3.5 Comparison of patient satisfaction

As shown in Table 5, there were differences in patient satisfaction levels between the two groups. In the observation group, 56.0% of patients reported being very satisfied and 40.5% were basically satisfied, while 3.6% were dissatisfied. In the control group, 51.9% were very satisfied, 28.2% were basically satisfied, and 19.9% were dissatisfied. The overall satisfaction rate was 96.4% in the observation group and 80.1% in the control group. Statistical analysis showed a significant difference in total satisfaction between the two groups (*χ*^2^ = 21.25, *p* < 0.001).

**Table 5:** Comparison of patient satisfaction between the control and observation groups.

Satisfaction Level	Observation Group (*n* = 168)	control group (*n* = 156)	*χ*^2^-value	*p*-value
Very satisfied	94 (56.0%)	81 (51.9%)	—	—
Basically satisfied	68 (40.5%)	44 (28.2%)	—	—
Dissatisfied	6 (3.6%)	31 (19.9%)	—	—
Total satisfaction	162 (96.4%)	125 (80.1%)	21.25	<0.001

### 3.6 Power analysis

A *post hoc* power analysis was conducted to evaluate whether the study had sufficient statistical power to detect differences in the primary and key secondary outcomes. The primary outcomes were resuscitation success rate and time to myocardial reperfusion. The key secondary outcomes included resuscitation room time and LVEF on day 3 after admission. Based on the observed effect sizes and the group sample sizes (observation group, *n* = 168; control group, *n* = 156), the calculated statistical power (1 − *β*) exceeded 98% for all endpoints. Specifically, the power was 99.99% for reperfusion time (Cohen's *d* = 0.630), 98.4% for resuscitation success rate (Cohen's *h* = 0.457), 99.99% for resuscitation room time (Cohen's *d* = 0.632), and approximately 100% for LVEF on day 3 (Cohen's *d* = 0.952). These findings confirm that the study was well powered (≥80%) to detect clinically meaningful differences between groups with high statistical confidence.

## 4 Discussion

This study examined the impact of a fast-track nursing pathway on clinical outcomes in patients with AMI undergoing emergency PCI (10, 11). The findings demonstrated that implementation of a structured, nurse-led fast-track pathway significantly improved emergency treatment efficiency, promoted early recovery of left ventricular function, and increased patient satisfaction, without elevating the risk of in-hospital complications. Baseline demographic and clinical characteristics were comparable between the observation and control groups, ensuring the reliability of outcome comparisons. The absence of significant differences in demographics, comorbidities, and illness severity, as measured by APACHE II scores, further supports the internal validity of the study. A key finding was the marked improvement in emergency treatment metrics in the observation group. The fast-track pathway significantly reduced both resuscitation room time and time to myocardial reperfusion. These time-sensitive indicators are clinically critical, as prolonged ischemia is closely associated with increased myocardial injury, adverse ventricular remodeling, and higher mortality. The observed reduction in these intervals through protocol-driven nursing interventions likely contributed to the higher resuscitation success rate in the intervention group.

In terms of cardiac recovery, both groups showed improvement in LVEF by day 3, but the increase was significantly greater in the observation group, suggesting that earlier reperfusion and coordinated nursing interventions accelerated myocardial functional recovery. LVEF is a critical prognostic marker following AMI and strongly correlates with both short- and long-term outcomes (12). In contrast, LVESV and LVEDV did not differ significantly, likely due to the short observation period, as ventricular remodeling typically requires longer follow-up (13, 14). The incidence of adverse events was low and comparable between groups (7.7% in both), indicating that the fast-track nursing pathway improved care efficiency without compromising patient safety. Although arrhythmias were slightly more frequent in the observation group, this difference was not statistically significant and may be attributed to closer monitoring and earlier intervention rather than a true increase in risk. Moreover, no cases of cardiogenic shock or cardiac rupture were reported in either group, further confirming procedural safety. Patient satisfaction was significantly higher in the observation group (96.4% vs. 80.1%), suggesting that structured communication, reduced delays, and proactive nursing engagement enhanced the patient care experience. High satisfaction levels are clinically relevant, as they are associated with improved adherence, better recovery, and reduced complaint rates (15, 16). Finally, a *post hoc* power analysis demonstrated that the study was adequately powered to detect clinically meaningful differences in all key endpoints, supporting the robustness and reliability of the findings.

Previous studies have explored strategies to enhance the efficiency of emergency management in AMI, yet few have emphasized the independent contribution of nursing-led interventions (2, 17, 18). Gopinath et al. (19) reported that system-level quality improvement measures in high-volume emergency departments significantly reduced door-to-balloon times among STEMI patients, primarily through organizational workflow optimization. Consistent with their findings, our study confirms that similar improvements can be achieved through a nurse-led, structured fast-track nursing pathway, underscoring the critical role of nursing coordination in accelerating reperfusion and improving rescue success. Zhang et al. (20) proposed a randomized controlled trial protocol assessing the impact of a clinical nursing pathway on PCI outcomes, anticipating gains in efficiency, safety, and satisfaction. Our findings provide empirical evidence supporting this conceptual framework, demonstrating that a structured fast-track model effectively enhances reperfusion timeliness, early ventricular function recovery, and patient satisfaction, thereby filling an existing evidence gap. Furthermore, Saban et al. (21) showed that fast-track interventions reduced emergency department delays but highlighted persistent sociodemographic disparities in care delivery. In contrast, our results indicate that standardized, nurse-led protocols can achieve consistently improved outcomes across patient groups, independent of socioeconomic confounders (22). Collectively, these comparisons reinforce that optimizing nursing processes is a practical, scalable, and safe approach to improving clinical efficiency and patient-centered outcomes in emergency PCI for AMI (23, 24).

This study provides new evidence on the effectiveness of a nurse-led, structured fast-track nursing pathway specifically designed for emergency PCI in patients with AMI. While previous studies have emphasized the importance of reducing door-to-balloon (D2B) time, few have systematically evaluated the independent contribution of nursing interventions to this process. The present study uniquely demonstrates that optimizing nursing coordination from triage through catheterization can independently shorten reperfusion time, improve early cardiac function recovery, and enhance patient satisfaction without increasing complications. This highlights the critical yet often underrecognized role of nursing leadership and process innovation in acute cardiovascular care. The findings underscore that structured nursing protocols are not merely supportive but clinically transformative, directly influencing key outcomes such as reperfusion efficiency and early ventricular function recovery. The significant improvement in rescue success rate (94% vs. 79%) and LVEF restoration within just three days demonstrates that a well-coordinated nursing system can enhance myocardial salvage and functional recovery during the most critical phase of AMI management. The fast-track nursing pathway developed in this study provides a replicable and scalable model for emergency cardiac care systems. By integrating standardized workflows, real-time communication, and designated team roles, this model ensures rapid transition from emergency admission to revascularization. Its implementation can be adapted across institutions to reduce treatment delays, optimize resource allocation, and improve both clinical efficiency and patient-centered outcomes. Moreover, the demonstrated safety profile supports the broader adoption of nurse-led fast-track protocols as part of quality improvement initiatives in cardiovascular emergency management. The results of this study support the integration of fast-track nursing pathways into standard emergency care protocols for AMI (25). Streamlined nursing workflows can reduce treatment delays, improve early cardiac recovery, and enhance patient satisfaction without increasing the risk of adverse events. Such pathways may be especially beneficial in high-volume centers or healthcare systems with limited physician availability during off-hours (26).

### 4.1 Strengths and limitations

This study has several strengths. First, the relatively large sample size and well-balanced baseline characteristics between groups enhance the reliability and validity of the results. Second, the inclusion of objective clinical endpoints, such as reperfusion time and LVEF, provides a robust evaluation of the intervention's effectiveness. Third, incorporating patient satisfaction outcomes offers a valuable patient-centered dimension to the analysis. However, several limitations should be acknowledged. This was a single-center retrospective study, which may limit the generalizability of the findings. The relatively short observation period precluded assessment of long-term outcomes, such as rehospitalization and mortality. Additionally, satisfaction data were collected using self-developed questionnaires, which, despite demonstrating internal consistency, may have introduced measurement bias. Future research should employ multicenter prospective designs, longer follow-up durations, and validated instruments to enhance the robustness and applicability of the findings.

## 5 Conclusions

The implementation of a fast-track nursing pathway for emergency PCI in patients with AMI significantly improved treatment efficiency, enhanced early recovery of left ventricular function, and increased patient and patient satisfaction, without elevating the risk of adverse events. These findings support the clinical value of nurse-led process optimization in the acute management of AMI.

## Data Availability

The raw data supporting the conclusions of this article will be made available by the authors, without undue reservation.
